# Magnetic resonance imaging evidence of hippocampal structural changes in patients with primary biliary cholangitis

**DOI:** 10.1038/s41424-018-0038-z

**Published:** 2018-07-06

**Authors:** Victoria A. L. Mosher, Mark G. Swain, Jack X. Q. Pang, Gilaad G. Kaplan, Keith A. Sharkey, Glenda M. MacQueen, Bradley G. Goodyear

**Affiliations:** 1 Seaman Family MR Research Centre, University of Calgary, Calgary, AB Canada; 2 Department of Medicine, University of Calgary, Calgary, AB Canada; 3 Snyder Institute for Chronic Diseases, University of Calgary, Calgary, AB Canada; 4 Liver Unit – Calgary Division of Gastroenterology and Hepatology, University of Calgary, Calgary, AB Canada; 5 Hotchkiss Brain Institute, University of Calgary, Calgary, AB Canada; 6 Department of Physiology and Pharmacology, University of Calgary, Calgary, AB Canada; 7 Department of Psychiatry, University of Calgary, Calgary, AB Canada; 8 Mathison Centre for Mental Health Research and Education, University of Calgary, Calgary, AB Canada; 9 Department of Radiology, University of Calgary, Calgary, AB Canada; 10 Department of Clinical Neurosciences, University of Calgary, Calgary, AB Canada

## Abstract

**Introduction:**

Behavioral symptoms are commonly reported by patients with primary biliary cholangitis (PBC). In other patient populations, symptoms are commonly associated with hippocampal volume reduction linked to neuroinflammation (inferred from regional iron deposition), as demonstrated by magnetic resonance imaging (MRI). We hypothesized that PBC patients would exhibit reduced volume and increased iron deposition of the hippocampus.

**Methods:**

Seventeen female non-cirrhotic PBC patients and 17 age/gender-matched controls underwent 3-Tesla T_1_-weighted MRI and quantitative susceptibility mapping (QSM; an indicator of iron deposition). The hippocampus and its subfields were segmented from T_1_ images using *Freesurfer*, and susceptibility of the whole hippocampus was calculated from QSM images. Volume and susceptibility were compared between groups, and associations with PBC-40 score and disease indicators (years since diagnosis, Fibroscan value, alkaline phosphatase level, clinical response to ursodeoxycholic acid (UDCA)) were investigated.

**Results:**

PBC patients exhibited significantly reduced hippocampal volume (*p* = 0.023) and increased susceptibility (*p* = 0.048). Subfield volumes were reduced for the subiculum, molecular layer, granule cell layer of the dentate gyrus and CA4 (*p* < 0.05). Fibroscan value was significantly correlated with PBC-40 (Spearman’s rho = 0.499; *p* = 0.041) and disease duration (Spearman’s rho = 0.568; *p* = 0.017).

**Discussion:**

Our findings suggest hippocampal changes occur early in the disease course of PBC, similar in magnitude to those observed in major depressive disorder and neurodegenerative diseases.

**Translational impact:**

Clinical management of PBC could include early interventional strategies that promote hippocampal neurogenesis that may beneficially impact behavioral symptoms and improve quality of life.

## Introduction

Primary biliary cholangitis (PBC) is an autoimmune liver disease characterized by inflammatory destruction of the hepatic interlobular bile ducts. PBC can progress to cirrhosis, liver failure and death or liver transplantation within 10 to 20 years^1^. Ursodeoxycholic acid (UDCA) delays disease progression in some patients^2^, but it does not alleviate commonly reported behavioral symptoms^3–5^ including fatigue^6–8^, memory/concentration problems^9^ and depressed mood^10,11^. Although these symptoms are typically unrelated to disease severity^7,12^, animal models of cholestatic liver disease strongly suggest they have a neurological basis^13^, and are thus not merely emotional reactions to having a chronic immune-mediated inflammatory disease.

Even though behavioral symptoms significantly impact quality of life^14^, their prevalence in PBC patients remains unclear. Based on questionnaires, it is estimated that 20–45% of PBC patients experience depression^7^; however, clinical interview estimates only 4.2% of PBC patients are actually depressed^15^. The prevalence of cognitive dysfunction in PBC patients also remains unclear; one study suggests that up to 80% of PBC patients experience some degree of cognitive deficit, with 53% of patients reporting their cognitive symptoms as moderate to severe^9^. The significant impact of memory and concentration deficits in PBC is also highlighted in the PBC-40 questionnaire, as the cognitive questions on this questionnaire mainly target deficits associated with memory and concentration^16^.

In other patient populations, depressive symptoms have been linked to dysfunction of the brain’s limbic network^17,18^. The hippocampus is a major component of the limbic system, and is involved in learning, memory and mood regulation^19,20^. In patients with major depressive disorder (MDD), reduction in hippocampal volume has been observed using neuroimaging^21,22^. The hippocampus may be particularly vulnerable because it is the only region of the adult brain involved in neurogenesis^23^. It is thought that new cells play a role in cognition and brain repair, as interventions that increase neurogenesis are associated with improved memory and increased synaptic plasticity^24^. Indeed, hippocampal neurogenesis is required to achieve the beneficial behavioral effects of antidepressants in patients with MDD^25^. The hippocampus can be subdivided into subfields that perform specific functions, and reduced subfield volume has been reported for the dentate gyrus^26,27^ and CA1-3^26^ in MDD patients. Although the cellular correlates of reduced hippocampal volume are not clearly known, post-mortem studies suggest reductions in neuropil^28^, granule neurons of the dentate gyrus^29^, and astrocyte density^30^ as potential sources. A loss of neurogenesis within the dentate gyrus of the hippocampus has also been suggested^31^.

Brain region volume loss has also been reported in the context of systemic inflammation^32^ and neuroinflammation^33^. One consistently reported consequence of neuroinflammation is an accumulation of iron. Typically, iron levels within the brain are tightly regulated, as both a deficiency and accumulation of brain iron can be detrimental. Under normal circumstances, macrophages (including microglia, the macrophage of the brain) use iron to produce free radicals in order to destroy pathogens; however, excess iron can lead to the generation of free radicals and tissue damage^34^. It is hypothesized that disruption within iron metabolism pathways in the brain is responsible for iron accumulation in neurodegenerative diseases^35,36^; however, specific mechanisms of iron accumulation in the brain remain unknown. Furthermore, the accumulation of iron could lead to oxidative stress and thus impact function, such as neurogenesis within the hippocampus.

Quantitative susceptibility mapping (QSM) is an MR imaging technique that allows for in vivo quantification of iron^37^, based on the degree to which iron distorts the local magnetic field (i.e., increases magnetic susceptibility). Accumulation of iron within the hippocampus is associated with normal aging^38^, and iron is present in excess amounts in a number of neurological diseases including multiple sclerosis (MS)^39^ and Alzheimer’s Disease^40^. Interestingly, in MS patients, increased susceptibility of the basal ganglia has been observed in tandem with reduced tissue volume^41^, suggesting the two may be linked. Increased iron deposition has also been linked to reduced cognitive ability^42^. To date, the association between hippocampal volume, hippocampal susceptibility, mood and cognitive ability has not been established; however, evidence from MDD studies suggests a strong correlation between hippocampal volume and cognitive dysfunction^43^.

Our recent resting-state functional magnetic resonance imaging (fMRI) study demonstrated altered functional connections of the hippocampus in patients with PBC^44^. The strength of functional connections between the hippocampus and brain regions involved in cognition and emotional processing was increased in PBC patients, compared to healthy controls. The present study builds on these previous findings; we re-examined a subset of our PBC patients who also underwent volumetric MR imaging and QSM, to specifically investigate the impact of PBC on the structure of the hippocampus. Given the overlap of PBC-associated behavioral symptoms with those observed in MDD patients and other neurological diseases, we hypothesized that PBC patients exhibit decreased volume and increased susceptibility of the hippocampus. We also investigated associations between hippocampus volume/susceptibility and disease duration, disease indicators, PBC-40 score, depression and clinical response to UDCA.

## Methods

This study was approved by the Conjoint Health Research Ethics Board of the University of Calgary, and written informed consent was obtained from all participants prior to their participation. Seventeen female patients with PBC (age range = 38–72, median = 53, IQR = 9) and seventeen female age-matched healthy control volunteers (age range = 42–63, median = 53, IQR = 5.5) underwent volumetric MRI and QSM, using 3 Tesla GE Discovery MR750 scanner equipped with a 12-channel receive-only phased array head coil (GE Healthcare, Waukesha, WI). PBC patients were consecutively recruited from the University of Calgary Liver Unit. All patients were taking UDCA for at least six months (mean dose 15.0 mg/kg/day) and met the standard criteria for the diagnosis of PBC, including anti-mitochondrial antibody (AMA) positivity and abnormal cholestatic liver biochemistry prior to the initiation of UDCA. Patients were identified as responders or non-responders to UDCA, where complete UDCA response was defined as a sustained normalization of serum alkaline phosphatase levels^45^. Non-cirrhotic, early disease patients were intentionally selected based on biochemistry (i.e., normal INR, and normal serum bilirubin and albumin concentrations) and liver stiffness (<16.9 kPa^46^) as measured by transient elastography (Fibroscan^®^; Echosens, Paris, France). Patients were excluded if they had significant medical comorbidities (diabetes, neurological disease, previous diagnosis of mood disorder, cardiac, or respiratory disease) or contraindications to MRI. Patient demographic and clinical characteristics are shown in Table 1.

**Table 1:** Patient demographic and clinical characteristics

Patient	Age (years)	Years since diagnosis (years)	Fibroscan value (kPa)	Alkaline phosphatase (U/L)	PBC-40 score	UDCA complete responder	HAM-D
1	52	2	3.0	162	41	Yes	1
2	38	2	6.9	132	52	No	3
3	60	8	4.0	121	65	Yes	1
4	72	8	3.7	251	43	No	0
5	59	14	8.9	89	57	Yes	2
6	53	2	4.0	122	47	Yes	0
7	54	15	13.4	208	128	No	1
8	64	8	4.8	183	93	No	1
9	60	4	4.3	154	64	No	2
10	53	10	8.6	100	102	Yes	13
11	53	6	8.0	113	54	No	5
12	68	9	4.8	147	70	No	0
13	46	6	11.6	189	45	No	0
14	58	9	14.1	217	114	No	1
15	53	4	4.3	35	47	Yes	0
16	47	1	7.0	143	107	Yes	3
17	50	1	3.5	85	93	Yes	1
Median	53	6	4.8	143	64	—	1

Within 24 h of MRI, all PBC patients completed the Hamilton Depression Rating Scale (HAM-D) and the PBC-40 questionnaire (see Table 1). The PBC-40 questionnaire is a well-validated PBC-specific survey designed to assess the impact of fatigue, pruritus, and general symptoms, as well as emotional, cognitive, and social function on the quality of life of PBC patients^12,16^.

For volumetric imaging, high-resolution anatomical MR images were collected using a three-dimensional magnetization-prepared rapid gradient-recalled echo imaging sequence (inversion/repetition/echo time = 550/8.2/3.2 ms, 0.8 × 0.8 × 1.3 mm voxels), and were processed using *FreeSurfer* version 6.0^47^ to automatically segment the hippocampus and its subfields^48^. Briefly, *Freesurfer* applies motion correction, removes non-brain tissue, registers the images to Talairach coordinates, tessellates white and gray matter boundaries, and applies topology correction and surface deformation (for details see refs. ^49,50^). Hippocampal and subfield volumes were calculated and used in a repeated-measures general linear model (GLM) with hemisphere (left or right) as the within-subject variable, group (PBC or healthy control) as the between-subject factor, and age and total intracranial volume as covariates.

QSM data were collected using an RF-spoiled, flow-compensated 3D gradient echo sequence (repetition/echo time = 29.5/26.3 ms; flip angle = 20°; FOV = 256 × 256 × 132 mm^3^; voxel size = 1 × 1 × 1 mm^3^, 8 echoes). QSM data from two PBC patients were lost due to technical issues during the session. QSM images were generated using *Cerebra-QSM* (Calgary Image Processing and Analysis Centre, Calgary, AB). The structural images obtained from this imaging sequence (i.e., magnitude images) were used to spatially register the QSM images to the anatomical images, and average susceptibility was calculated for each of the left and right hippocampus, as segmented by *Freesurfer*. Obtaining average susceptibility for each hippocampus subfield was not possible due to the spatial smoothness of the final QSM images. Average susceptibility of the hippocampus was used in a repeated-measures GLM, with hemisphere (left or right) as the within-subject variable, group (PBC and healthy controls) as the between-subject variable and age as a covariate.

For patient data, Pearson correlation analyses (or Spearman non-parametric, where appropriate) were conducted to determine any significant associations with HAM-D, PBC-40 score, Fibroscan^®^ value, alkaline phosphatase level, and disease duration. An additional ANOVA was performed to determine if volume or susceptibility differed between UDCA responders and non-responders.

## Results

**Fig. 1:**
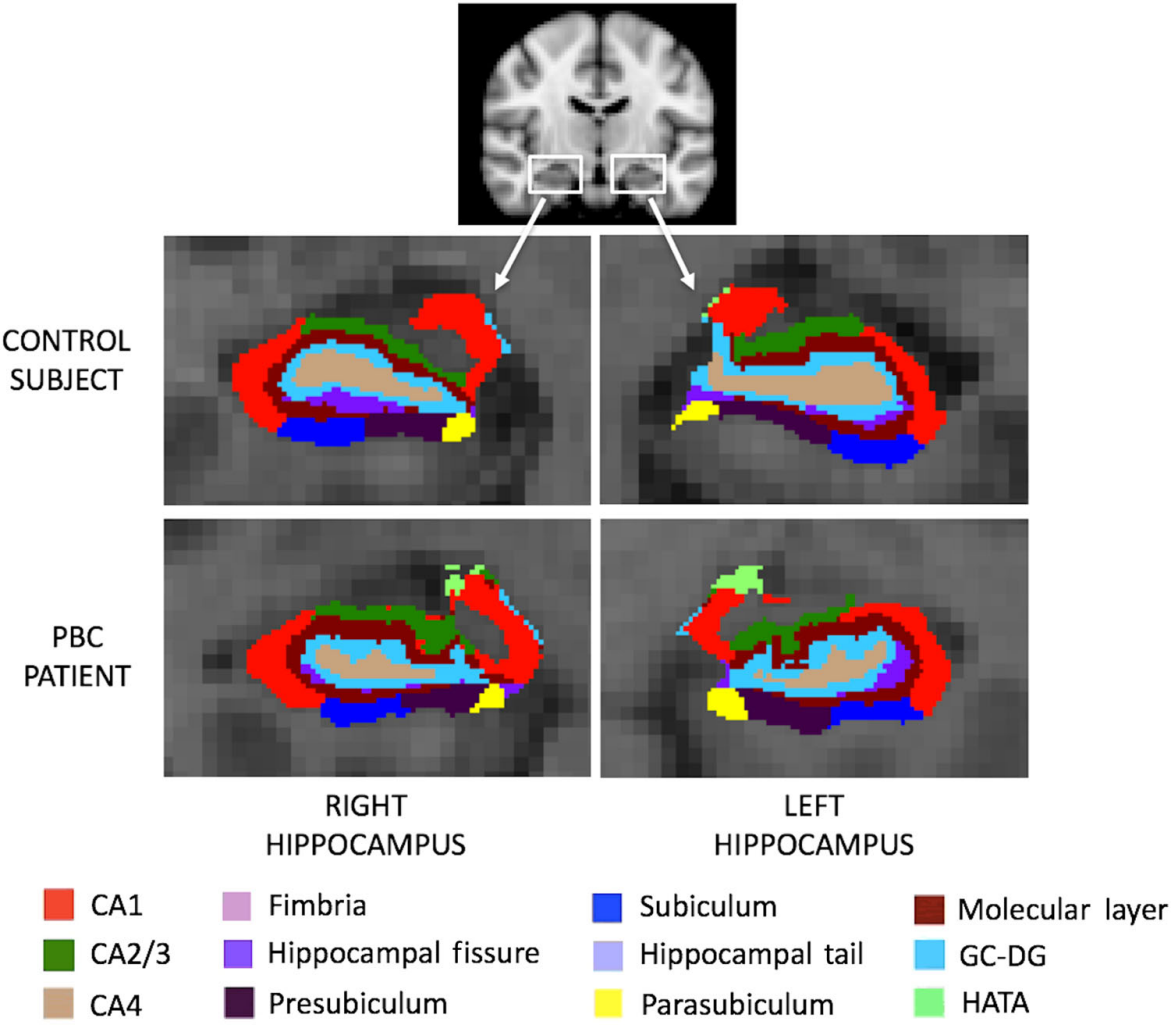
Representative segmentation and subfields of the left and right hippocampus for an individual control subject and PBC patient

**Fig. 2:**
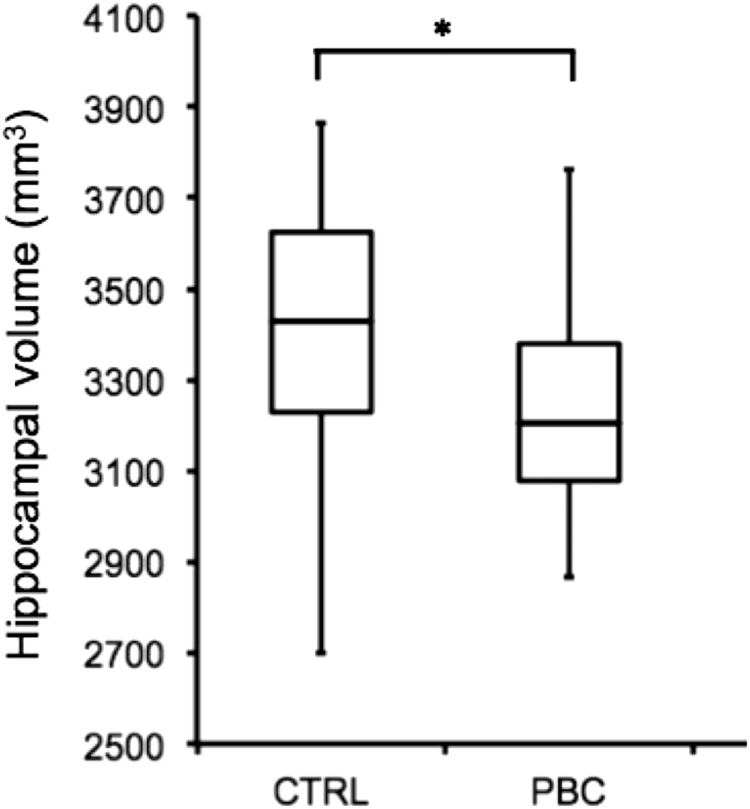
Hippocampal volume (in mm^3^) was significantly reduced in PBC patients as compared to healthy controls (*p = 0.023)

**Fig. 3:**
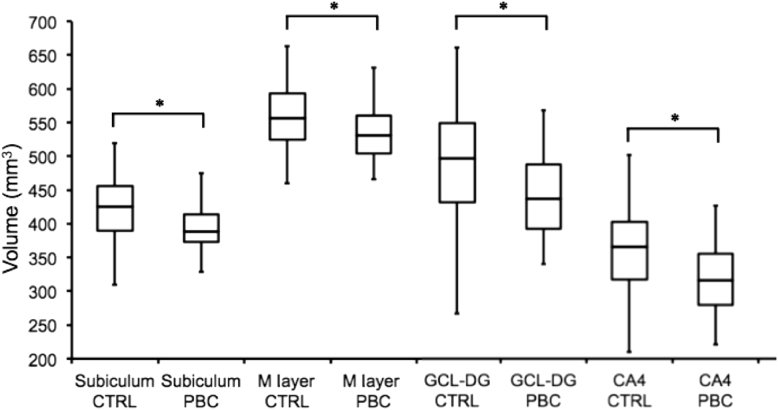
Volume of the subiculum (*p* *=* 0.029), molecular layer (M Layer; *p* *=* 0.018), granule cell layer of the dentate gyrus (GCL-DG; *p* *=* 0.035) and CA4 (*p* = 0.005) were significantly reduced in PBC patients as compared to healthy controls (*p < 0.05)

Representative hippocampal subfield segmentations are shown in Fig. 1. Segmentations were successful for all participants. There was a significant main effect of group on hippocampal volume [*F*(1,32) = 5.733, *p* = 0.023]; hippocampal volume was significantly less in PBC patients, relative to controls (Fig. 2). Analysis of subfield volumes revealed a significant main effect of group on the volumes of the subiculum [F(1,32) = 5.268, *p* = 0.029], molecular layer [*F*(1,32) = 6.319, *p* = 0.018], granule cell layer of the dentate gyrus [*F*(1,32) = 4.894, *p* = 0.035], and CA4 [*F*(1,31) = 9.153, *p* = 0.005]; volumes were significantly less in PBC patients, relative to controls (Fig. 3). As only one PBC patient had a HAM-D score greater than 7 to indicate mild depression, no associations between volumes and HAM-D score were investigated. For PBC patients, volumes did not correlate with any of PBC-40 score, Fibroscan^®^ value, alkaline phosphatase level or disease duration.

**Fig. 4:**
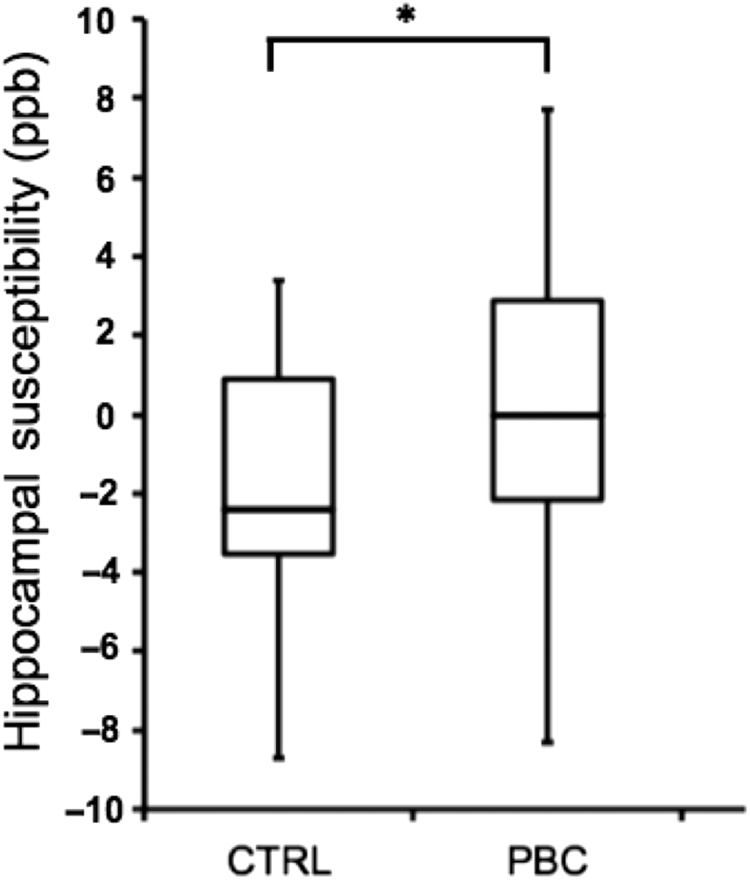
In comparison with healthy controls (CTRL), PBC patients exhibited increased susceptibility (in parts per billion) of the hippocampus (*p = 0.048)

There was a significant main effect of group on hippocampal susceptibility [*F*(1,29) = 4.26, *p* = 0.048]; hippocampal susceptibility was significantly greater in PBC patients, relative to controls (Fig. 4). For PBC patients, there were no significant correlations with PBC-40 score, Fibroscan^®^ value, alkaline phosphatase level or disease duration. No association between susceptibility and HAM-D score was investigated.

Hippocampal susceptibility also did not correlate with hippocampal volume when controlling for age. PBC-40 score was significantly correlated with Fibroscan value (Spearman’s rho = 0.499; *p* = 0.041) and disease duration (Spearman’s rho = 0.568; *p* = 0.017).

## Discussion

We have shown that the hippocampus exhibits reduced volume in patients with PBC, relative to healthy control subjects. Additionally, we have shown that these PBC-associated hippocampal volume changes are in turn paralleled by evidence of increased neuroinflammation or oxidative stress (as indicated by increased iron deposition in the hippocampus, reflected by enhanced susceptibility). However, neither hippocampal volume nor susceptibility were associated with any disease indicator.

Further, we observed reduced volume of specific subfields of the hippocampus in PBC patients relative to healthy controls (subiculum, granule cell layer of the dentate gyrus, layer CA4 and molecular layer). The subiculum is the main output region of the hippocampus, and is thought to be involved in spatial navigation, memory, and regulation of the hypothalamic–pituitary–adrenal (HPA) axis^51,52^. The granule cell layer of the dentate gyrus is thought to be involved in spatial memory, and neurogenesis in this region can be inhibited by stress^53,54^. CA4 is, in fact, a layer of the dentate gyrus^55^, and the molecular layer encompasses the CA fields as well as the dentate gyrus^56^. It has been reported that the functions of both the subiculum and the dentate gyrus decline with age, with further decreases associated with memory decline^57^.

Strikingly, the hippocampal volume reduction observed in our PBC patients was similar in magnitude to that observed in previous studies of patients with MDD^22^. There is an increasing appreciation that MDD is associated with the presence of a systemic inflammatory response^58^. Systemic inflammation, associated with hippocampal neurodegeneration and depressive symptoms^59^, is also evident in a number of neurological diseases including Alzheimer’s disease^60^ and multiple sclerosis (MS)^61^. Indeed, animal models of systemic inflammatory states identify depressive-like symptoms associated with decreased hippocampal volume^62,63^. However, only one PBC patient exhibited any significant signs of depression (the data from this patient did not act as an outlier for any measure). Thus, our observations are clearly not simply the result of depression. However, the similarities between MDD and PBC in terms of hippocampal volume reduction suggest a common underlying mechanism.

As the median disease duration for our patients was only 6 years, these changes must occur early in the disease process. It is possible that during the early stages, hippocampal volume reduction reflects the degradation of the supportive structure (e.g., neuropil) rather than a loss of neurons, and as a result, behavioral symptoms are not yet present. One could postulate that as the disease progresses, neurons eventually become lost, leading to symptom onset. It is important to note that the patients in the present study are a subset of the patients we previously investigated using resting-state fMRI^44^. In that paper we reported that PBC patients exhibited lower scores than controls on a test of verbal working memory, but not on tests of spatial working memory, visual attention, and task switching. As these cognitive domains are not directly related to hippocampal function, test scores were not investigated in the present study; no associations were confirmed in follow-up analyses. Other cognitive domains were not tested. Thus, there exists the possibility that we may have missed hippocampus-related behavioral symptoms.

Interestingly, there were no correlations between clinical characteristics and hippocampal volume or susceptibility. This observation may also suggest that disease-associated decreases in hippocampal volume and increases in iron deposition occur very early during PBC disease natural history, before classical diagnostic definitions for PBC have been met. That is, once PBC has been diagnosed, the hippocampal changes are already present. If this is the case, then increased population screening to identify people at risk of developing PBC may be justified, in order to initiate therapies to prevent hippocampal structural changes. One possible screen is AMA positivity, with no other signs of liver disease. The presence of AMAs is a highly disease-specific biomarker, with 90–95% of patients and less than 1% of healthy normal controls exhibiting elevated AMAs^64,65^. Interestingly, in healthy controls with elevated AMAs, liver biopsies suggest that a significant proportion of these individuals have histological inflammatory changes consistent with PBC^66^, and many of these individuals go on to develop PBC^67^.

Hippocampal volume and susceptibility also did not differ between UDCA responders and non-responders. This could be due to the low numbers in these subgroups. Alternatively, it is possible that UDCA did have an impact within the group that responded; however, pre-treatment MRI would be necessary to observe a treatment effect.

Inflammation is an important driver of neurodegeneration^68^ and is associated with a variety of neurological diseases, including Alzheimer’s disease^69^. Alzheimer’s disease is characterized by extensive volume loss of the hippocampus, as well as memory deficits and cognitive decline^70^. In a mouse model of Alzheimer’s disease, injection of bacteria to induce systemic infection increases hippocampal-dependent cognitive decline, as well as accelerates disease progression associated with microglia activation within the hippocampus^71^. This pattern of activated microglia within the hippocampus has also been found in post-mortem studies of patients with Alzheimer’s disease^72^. However, because hippocampal susceptibility and volume were not directly correlated in our patients, it is unlikely that neuroinflammation is solely responsible for volume reduction. Oxidative stress could also be factor.

Choosing clinically mild PBC patients generated a homogeneous sample that simplified group analyses and allowed us to exclude brain changes associated with advanced liver fibrosis and cirrhosis, including subclinical hepatic encephalopathy. However, it limited our ability to effectively elucidate any interrelationships between hippocampal volume reduction, hippocampal susceptibility increases, symptom severity, and disease indicators. This can be viewed as a study limitation. Future studies could include patients with greater symptom and disease burden in order to ascertain whether hippocampal abnormalities are indeed associated with symptom and disease severity, or are already present at diagnosis. Additionally, by investigating only PBC patients, we were unable to determine the specificity of our findings. Future research should aim to include patients with other cholestatic liver diseases or systemic inflammatory diseases in order to ascertain whether these findings are specific to PBC or are more generalized to liver or inflammatory disease.

Our findings have the potential to foster new approaches for therapeutic intervention. For example, hippocampal neurogenesis (the failure of which is strongly associated with mental disorders^31^) is recognized as a promising target for the treatment of mental disorders. Exercise has been found to be an effective catalyst for neurogenesis within the hippocampus^73^, and can be an effective treatment for mental disorders, including MDD^74^ and Alzheimer’s Disease^75^. Therefore, exercise may also be useful as therapy for PBC patients to prevent or attenuate hippocampal changes and behavioral symptom development. Another way to potentially increase hippocampal neurogenesis in PBC patients is by taking advantage of the promotion of neurogenesis provided by antidepressants. Animal models suggest that the beneficial behavioral effects of antidepressants and neurogenesis are closely linked^25,76^. If antidepressant-related neurogenesis is prevented, the beneficial behavioral effects of the antidepressant are blocked^25^. Importantly, chronic treatment with antidepressants is necessary to increase hippocampal neurogenesis, which follows the time-course of the therapeutic actions of antidepressants in MDD patients^76^. While our PBC patients did not exhibit depressive-live symptoms, studies have shown positive effects of antidepressants on inflammatory bowel disease patients who do exhibit symptoms, though, efficacy was difficult to evaluate^77^. Antidepressants have also been suggested as a possible treatment for fatigue in PBC patients^78^; however, one small study did not show a benefit, and depressive symptoms were not assessed^79^. Larger placebo-controlled studies are necessary to determine the potential impact of antidepressants on improving hippocampal changes associated with PBC, and if these are in turn linked to symptomatic benefits. Clearly, it is through a better understanding of the pathogenesis of behavioral symptoms and their brain correlates in PBC patients that we may be able to develop treatments that can alleviate symptom burden experienced by PBC patients and improve their quality of life.

## Study highlights

### What is current knowledge?

- Fatigue, cognitive impairment, and altered mood significantly impact quality of life of many PBC patients.
- These symptoms have often been labeled as emotional reactions, and have therefore been often ignored.
- In non-hepatic diseases, symptoms have been linked to altered structure and function of the hippocampus.

### What is new here?

- PBC patients exhibit reduced volume of the hippocampus and some of its subfields.
- PBC patients exhibit increased iron deposition within the hippocampus.
- Hippocampal changes are neither correlated with markers of disease severity nor clinical response to therapy.
